# Dual-branch hybrid network for lesion segmentation in gastric cancer images

**DOI:** 10.1038/s41598-023-33462-y

**Published:** 2023-04-19

**Authors:** Dongzhi He, Yuanyu Zhang, Hui Huang, Yuhang Si, Zhiqiang Wang, Yunqi Li

**Affiliations:** 1 Faculty of Information Technology, Beijing University of Technology, Beijing, China; 2 Department of Gastroenterology, The First Medical Center, Chinese PLA General Hospital, Beijing, 100853 China; 3 Department of Gastroenterology, The Second Medical Center and National Clinical Research Center for Geriatric Diseases, Chinese PLA, Beijing, China

**Keywords:** Image processing, Gastrointestinal cancer, Network models

## Abstract

The effective segmentation of the lesion region in gastric cancer images can assist physicians in diagnosing and reducing the probability of misdiagnosis. The U-Net has been proven to provide segmentation results comparable to specialists in medical image segmentation because of its ability to extract high-level semantic information. However, it has limitations in obtaining global contextual information. On the other hand, the Transformer excels at modeling explicit long-range relations but cannot capture low-level detail information. Hence, this paper proposes a Dual-Branch Hybrid Network based on the fusion Transformer and U-Net to overcome both limitations. We propose the Deep Feature Aggregation Decoder (DFA) by aggregating only the in-depth features to obtain salient lesion features for both branches and reduce the complexity of the model. Besides, we design a Feature Fusion (FF) module utilizing the multi-modal fusion mechanisms to interact with independent features of various modalities and the linear Hadamard product to fuse the feature information extracted from both branches. Finally, the Transformer loss, the U-Net loss, and the fused loss are compared to the ground truth label for joint training. Experimental results show that our proposed method has an IOU of 81.3%, a Dice coefficient of 89.5%, and an Accuracy of 94.0%. These metrics demonstrate that our model outperforms the existing models in obtaining high-quality segmentation results, which has excellent potential for clinical analysis and diagnosis. The code and implementation details are available at Github, https://github.com/ZYY01/DBH-Net/.

## Introduction

Gastric cancer is one of the common malignant tumors, with more than 1 million new patients yearly^1^. The incidence of gastric cancer ranks among the top three cancers in China, with a mortality rate of 12.4%^2^. In terms of morbidity and mortality, gastric cancer is considered to be a severe and lethal malignancy^3^. Gastroscopy is the most common method of detecting and diagnosing gastric cancer. It highly relies on a great deal of expertise and practical experience by trained doctors. Research showed that the accuracy of manual gastroscopy is only 69–79%^4^. With the deep learning algorithms introduced into medical image segmentation, many studies have used Convolutional Neural Networks (CNNs) to segment gastric cancer images. Hirasawa et al.^5^ achieved automatic detection of gastric cancer in endoscopic images using CNNs, but the accuracy is limited due to ambiguous lesion features. PAN et al.^6^ identified early gastric cancer and non-cancerous images by improving the SSD model. The DSF module was proposed to achieve an effective fusion of features at different levels. ZHANG et al.^7^proposed an enhanced SSD architecture called SSD-GPNet. It takes advantage of the cross-layer relationship in the feature pyramid to increase the receptive field of the network and enhance feature extraction. Although using CNNs achieves a better recognition effect, the result can still not meet the requirements of complementary medical diagnosis. This prompted us to seek more targeted network structures to improve segmentation performance.

Ronneberger et al.^8^ proposed the U-Net in 2015, which uses skip connections to make the final restored feature map incorporate more low-level feature information and has wide application in medical image segmentation. Many studies have improved the U-Net to gastric cancer lesion segmentation. QIU et al.^9^ identified certain types of lesion sites in gastric cancer using an improved U-Net model based on pyramidal structure. ZHANG et al.^10^ developed a modified U-Net network that enhances the fusion of high-level and low-level feature information by designing SERES and DAGC modules to replace the pooling operation. Although the improved U-Net method has been proven more effective, its inherent limitations lead to its lack of capability in modeling explicit long-range relations. Due to the number of folds in gastric mucosa, the complexity of the gastric environment requires the model to have global information acquisition to distinguish lesion features from background noise. Therefore, the accurate and effective capture of global contextual information remains a crucial problem to be addressed.

Transformer^11^ is inherently good at modeling long-range dependencies, which focus on global contextual information and achieves better results in computer vision tasks^12–15^. Therefore, some research has attempted to apply the Transformer to image segmentation. Strudel et al.^16^ proposed a pure Transformer-based encoder-decoder architecture Segmenter, which captures global contextual information well and achieves excellent results in semantic segmentation. On the contrary, applying pure transformer in medical image segmentation leads to unsatisfactory training results, mainly due to the limited amount of data making it difficult to extract enough detailed information in the lowers layers. Therefore, the study proposed a strategy combining Transformer and CNNs in medical segmentation. Zhang et al.^17^ proposed the TransFuse model by combining Transformers and CNNs in a parallel manner to improve the efficiency of global context modeling without losing low-level features. CHEN et al.^18^ proposed the TransUNet model that recovers local spatial information by U-Net and allows the Transformer as an encoder for medical image segmentation. However, the model passes the deep features extracted by convolution into the Transformer, which makes the global information obtained by the Transformer fragmentary, and the advantage of the Transformer is fractional. Therefore, we propose to pass the original images into the Transformer and CNNs separately to capture the dominant features extracted by both fully, but this will undoubtedly lead to high model complexity. Low-level features contribute less to the performance of target segmentation in salient regions and increase computational complexity than deep-level features^19^. Hence, we need to design a decoder structure to replace the original one, eliminating the impact of low-level features on computational complexity. In addition, Transformer is based on global computational self-attention, which leads to computationally expensive. Liu et al.^20^ proposed Windows Multi-Head Self-Attention (W-MSA) in the Swin-Transformer to improve its self-attention calculation, significantly reducing the computational complexity.

Motivated by the above research, we propose a Dual-Branch Hybrid Network fusing Swin-Transformer and U-Net for gastric cancer image segmentation. We design a decoder structure that aggregates in-depth feature information to achieve accurate localization of lesion regions by Swin-Transformer and U-Net. In order to combine the extracted feature by U-Net and Transformer, we investigate an effective feature fusion technique. The multi-modal fusion mechanism^21^ enhances the extraction of correlation information at different scales. Moreover, the linear Hadamard product^22^ enables effective cross-fertilization of features. We calculate the Transformer loss, the U-Net loss, and the fused loss with the ground truth label for network training and finally output a high-quality segmentation result. In general, this work focuses on the following points.

- This paper proposes a Dual-Branch Hybrid Network to segment the lesions in gastric cancer images by fusing Swin-Transformer and U-Net in a parallel style.
- In this paper, we build the Deep Feature Aggregation Decoder (DFA) to replace the original decoders of Swin-Transformer and U-Net to reduce the complexity of the model and recover detailed information on the lesion regions.
- This paper constructs the Feature Fusion (FF) module, which can utilize the multi-modal fusion mechanisms to interact independent features of various modalities as well as the linear Hadamard product to fuse the features.
- We use professional evaluation metrics to assess the model. The experimental results show that the model can accurately segment the lesion region of gastric cancer images, and the results are better than the current state-of-the-art methods.

The remainder of this paper is organized as follows. Section "Related Works" presents the application of the improved U-Net and Transformer structure in medical image segmentation. Section "Method" describes the proposed framework in this study, including the Swin-Transformer branch, the U-Net branch, the DFA, FF, and Decoder modules, and the loss functions. The experimental results on several datasets are presented in Section "Experiments". Finally, we visualize the experimental results and list the conclusions.

## Related works

### U-shaped networks

The semantic structure of medical images is relatively simple, so their high-level semantic information and low-level features are essential. The U-Net has achieved a good performance in medical image segmentation by improving skip connection and providing more detailed information. Many variants of U-Net have achieved excellent performance. Oktay et al.^23^ proposed the Attention U-Net model, which incorporates integrated attention gates (AGs) to recalibrate the output features of the coding and effectively suppresses irrelevant noise to highlight the salient features of hopping connection delivery. Li et al.^24^ proposed an attention-based nested segmentation network, ANU-Net, which performs well on the liver tumor segmentation dataset LiTS by redesigning dense skip connections. Ni et al.^25^ proposed the RAUNet to solve the problem of specular reflection in cataract segmentation by adding an enhanced attention module to fuse multi-level features and capture contextual information effectively. MZ Alom et al.^26^ proposed the R2U-Net, which combines the advantages of U-Net, residual network, and RCNN network, and has better performance in retinal image segmentation tasks with the same number of parameters. ZHOU et al.^27^ proposed a segmentation architecture (UNet + +) based on nested dense skip connections, which demonstrates effectiveness on abdominal CT liver segmentation datasets and colonic polyp segmentation datasets. The above research confirms that the U-Net has become one of the most popular deep learning frameworks in medical image segmentation with good segmentation performance.

### Transformers applications

Transformer is widely used in many NLP tasks with good performance. The VIT (Vision Transformer) was first proposed by^28^ for image processing in 2020. It showed results comparable to the CNNs at that time but required significantly fewer computational resources to train. From then on, many studies have worked on solving medical image segmentation problems by using Transformer. Valanarasu et al.^29^ proposed the MedT model to solve the poor performance of Transformer on small medical datasets. It used a gated axial-attention model, which extends the existing architectures by introducing an additional control mechanism in the self-attention module. Ji et al.^30^ proposed the Multi-Composite Transformer (MCTrans), which integrated rich feature learning and semantic structure mining into a unified framework. Gao et al.^31^ proposed the UTNet, which applied self-attentive modules in the encoder and decoder to capture long-range dependencies with minimal overhead. Zhang et al.^32^ proposed Multi-Branch Hybrid Transformer Network (MBT-Net) based on a body-edge Branch to obtain more details and contextual information. Cao et al.^33^ proposed Swin-Unet, a U-shaped encoder-decoder structure based on Swin-Transformer blocks. It developed patch expanding layers to achieve up-sampling and feature dimensionality increase without convolution or interpolation operations. Lin et al.^34^ proposed DS-TransUNet to improve the problem of ignoring the intrinsic structural features at the pixel level during patch segmentation. Proposed TIF module to achieve efficient interaction at multi-scale features using MSA mechanism. The above studies confirm that Transformers are widely used in medical image segmentation and perform well.

## Method

**Figure 1:**
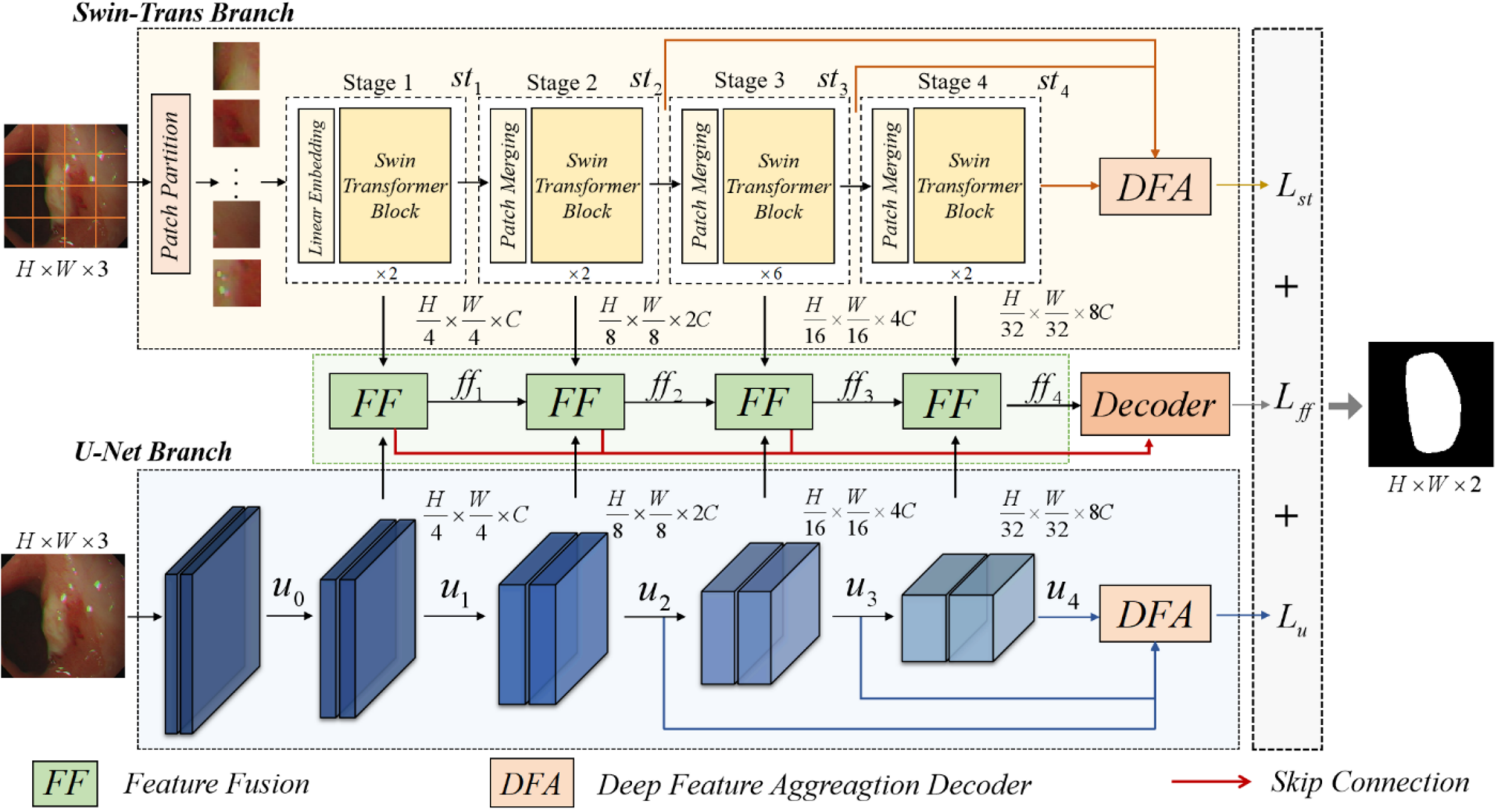
The overall framework of the proposed model. The structures of the DFA module, FF module and Decoder module are given in Figs. 3, 4, and 5, respectively; ${L}_{st},{L}_{u},{L}_{ff}$ represent the loss values of the U-Net branch, Swin-Transformer branch, and the fusion branch, respectively.

The overall framework of our proposed end-to-end Dual-Branch Hybrid Network is shown in Fig. 1. The U-Net branch extracts spatial information at each scale, and the Swin-Transformer branch captures global contextual information. To obtain the feature information of the salient lesion regions extracted from the two branches and reduce the complexity of the model, we propose the Deep Feature Aggregation Decoder (DFA) to aggregate the deep features to recover the spatial details of the lesion region and output the loss value between segmentation result and the ground truth label. In addition, the features extracted from both branches are fed into the Feature Fusion (FF) module for processing and passed into the Decoder module via a skip connection. The Decoder structure recovers the details of the image and the corresponding spatial dimensions to output the loss value and the final segmentation results. In addition, we combine the loss values obtained from the three components by weighting them for joint training to maximize the advantages of the two branches.

### Swin-transformer branch

The design of the Swin-Transformer branch follows the typical encoder-decoder architecture. In this case, the encoder architecture uses the Swin-Transformer architecture proposed in^20^. The decoder structure uses our proposed DFA module. The overall framework shown in the yellow dashed box in Fig. 1. Given an image $x\in {R}^{H\times W\times 3}$ with a spatial resolution of $H\times W$ and 3 number of channels. For the Swin-Transformer branch, the image is split into non-overlapping patches with a patch size of 4 $\times$ 4 in the Patch Partition module. Then the size of the image changes from $[H, W,3]$ to $[H/4,W/4,48]$. By linearly transforming the channel dimension of each pixel through the Linear Embedding layer, the number of channels changes from 48 to our pre-defined hyper-parameter $C$, flatten to $[H/4\times W/4,C]$. The transformed patch tokens are stacked with Swin-Transformer blocks and Patch Merging Layer to generate the hierarchical representation of the features. The Patch Merging layer is responsible for down-sampling and increasing dimension, while the Swin-Transformer block is responsible for feature representation learning. A total of 4 stages, setting the number of Swin-Transformer blocks in each stage to^2,2,2,6^. The feature maps obtained by down-sampling at each stage are passed into the FF module for the interactive fusion of information. The feature maps of the last three stages are passed into the DFA module to output the segmentation results. The final output feature map sizes are $[H/4,W/4,C]$, $[H/8,W/4,2C]$, $[H/16,W/16,4C]$, $[H/32,W/32,8C]$.

**Figure 2:**
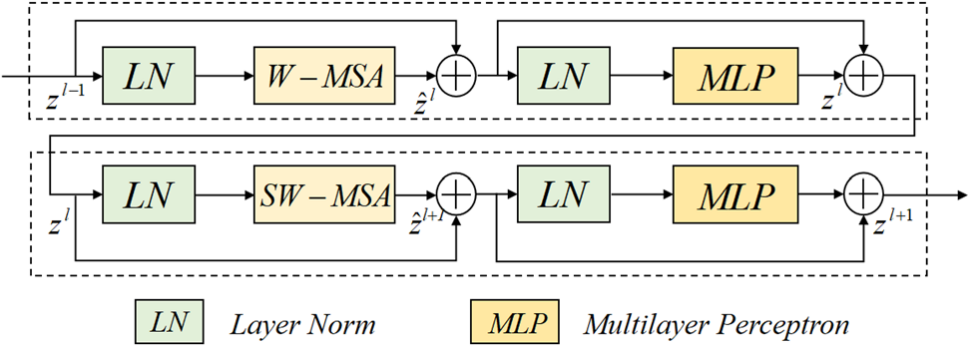
The diagram of Swin-Transformer Block.

The Transformer architecture uses the Multi-head Self Attention (MSA) module to compute global self-attention for feature learning, which results in computationally intensive and high model complexity. The Swin-Transformer block introduces the idea of local calculation, calculating self-attention in the window region without overlap, significantly reducing computational complexity. The general structure is shown in Fig. 2. Specifically, the Swin-Transformer block comprises two sets of Layer-Norm (LN) layers, the window-based MSA layer, a residual connection, and a 2-layer Multilayer Perceptron (MLP) unit. In this case, the window-based W-MSA module calculates the self-attention only for each window's interior. In contrast, the shifted window-based module (SW-MSA) is used to solve the problem of window-to-window information transfer. Based on such a window partitioning mechanism, the Swin-Transformer block can be formulated in Eqs. (1) to (4).

1 $$\begin{array}{*{20}c} {\tilde{z}^{l} = W - MSA\left( {LN\left( {z^{l - 1 } } \right)} \right) + z^{l - 1 } } \\ \end{array}$$

2 $$\begin{array}{*{20}c} {z^{l } = MLP\left( {LN\left( {\tilde{z}^{l} } \right)} \right) + \tilde{z}^{l} } \\ \end{array}$$

3 $$\begin{array}{*{20}c} {\tilde{z}^{l + 1} = SW - MSA\left( {LN\left( {z^{l } } \right)} \right) + z^{l } } \\ \end{array}$$

4 $$\begin{array}{*{20}c} {z^{l + 1 } = MLP\left( {LN\left( {\tilde{z}^{l + 1} } \right)} \right) + \tilde{z}^{l + 1} } \\ \end{array}$$

### U-Net branch

The encoder of the U-Net branch consists of 5 groups of convolutional units. Each set of convolution units uses a max-pooling layer with filter size 2 $\times$ 2 to halve the feature map size and two convolutional layers with kernel size 3 $\times$ 3 and a stride of 1 for feature extraction. After each convolution layer, there are a Batch Normalization (BN) layer and correction rectified linear unit (ReLU) activation functions. We input the feature maps extracted from the latter four groups of convolutional units into the FF module. The final output feature map sizes are $[H/4,W/4,C]$, $[H/8,W/4,2C]$, $[H/16,W/16,4C]$, $[H/32,W/32,8C]$.

The decoder uses our proposed DFA module to replace the original decoder in U-Net. The feature maps extracted from the last three convolutions are passed into the DFA module to output the segmentation results. The general structure is shown in the blue dotted box in Fig. 1. From experience, we set the value of hyper-parameter $C$ to 96.

### Deep feature aggregation decoder DFA

**Figure 3:**
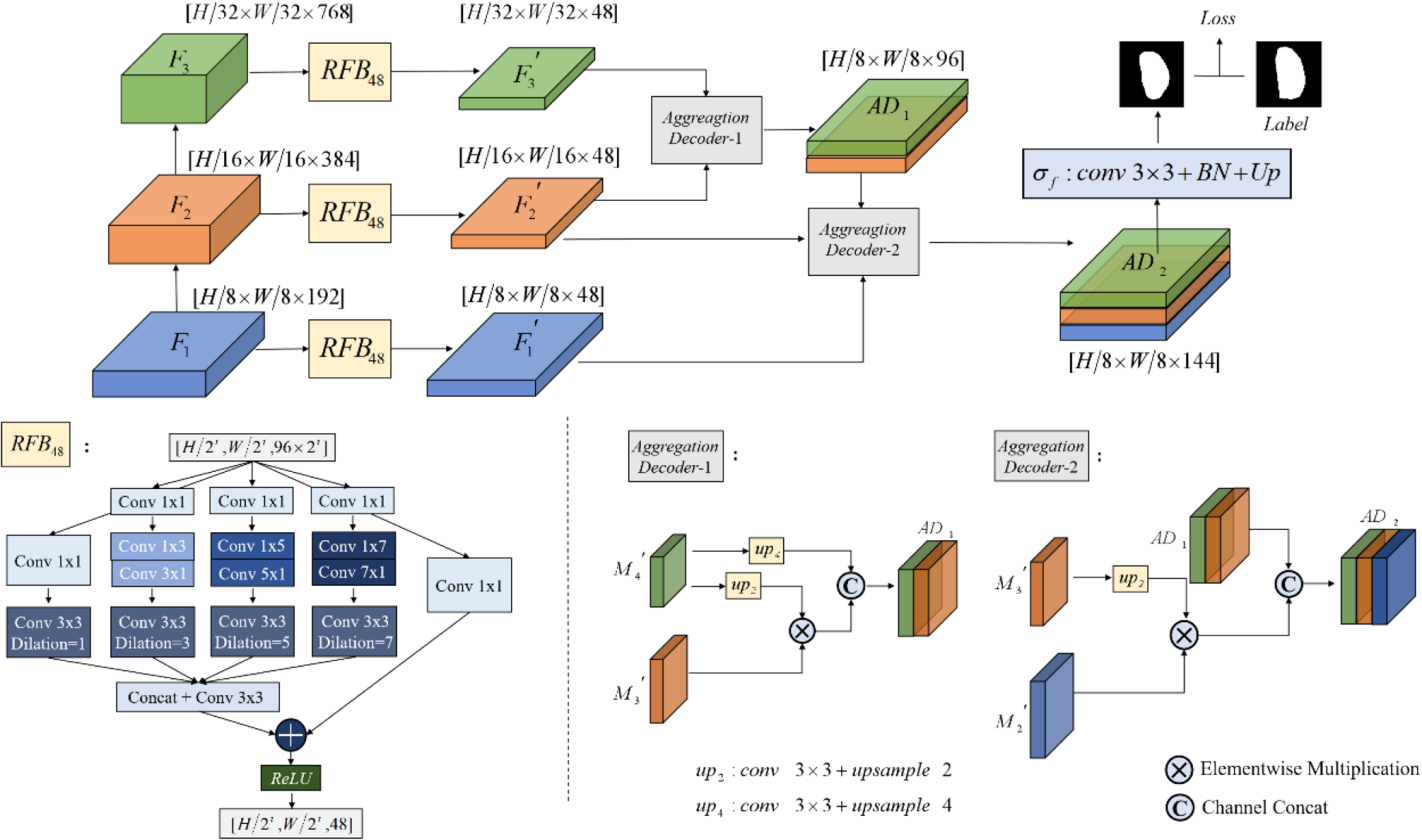
The diagram of the Deep Feature Aggregation Decoder DFA.

In order to output the segmentation results of the U-Net and the Swin-Transformer, we need to build a decoder structure to recover the image information. As we focus on the segmentation results of the Transformer and the U-Net for salient lesion regions, fast and accurate positioning is our primary objective. Therefore, to accurately locate the gastric cancer lesion region and reduce the complexity of the model, we propose the Deep Feature Aggregation Decoder (DFA) to eliminate the influence of low-level features on the computational complexity and recover the spatial detail of the lesion region. The structure of the module is shown in Fig. 3. We aggregate the output features of the last three modules ${F}_{i}, i=\mathrm{1,2},3$. In order to obtain global information on deeper features, we introduced the Receptive Field Block (RFB)^35^ to increase the receptive field. Compared to the conventional RFB module, we add a convolutional layer with a dilation rate of 7 and reduce the channel to 48 to decrease the computational loss of extracted features, as shown in module ${ RFB}_{48}$ in Fig. 3. We construct two aggregated feature decoders $Aggreagtion Decode{r}_{\mathrm{1,2}}$ to achieve the fusion of feature information at different scales. The structure is shown in Fig. 3. The decoder uses multiplication operation and concatenation in the channel dimension to feature interaction, and finally, a convolution layer with kernel size 3 $\times$ 3 and an interpolation layer with a $scale\_factor$ of 8 to obtain the final feature mapping. The formulation is shown in Eqs. (5) to (8).

5 $$\begin{array}{*{20}c} {F_{1}^{^{\prime}} , F_{2}^{^{\prime}} ,F_{3}^{^{\prime}} = RFB_{48} \left( {F_{1} ,F_{2} ,F_{3} } \right)} \\ \end{array}$$

6 $$\begin{array}{*{20}c} {AD_{1} = Channel\_Concat\left[ { Up_{2} \left( {F_{3}^{^{\prime}} } \right) \times F_{2}^{^{\prime}} , Up_{4} \left( {F_{3}^{^{\prime}} } \right) } \right]} \\ \end{array}$$

7 $$\begin{array}{*{20}c} {AD_{2} = Channel\_Concat\left[ { Up_{2} \left( {F_{2}^{^{\prime}} } \right) \times F_{1}^{^{\prime}} , AD_{1} } \right]} \\ \end{array}$$

8 $$\begin{array}{*{20}c} {{\text{Result}} = \sigma_{f} \left( {AD_{2} } \right)} \\ \end{array}$$

where ${Up}_{2}$ and ${Up}_{4}$ are linear interpolation operations with $scale\_factor$ of 2 and 4 respectively. ${\sigma }_{f}$ consists of two sets of convolutional layers with kernel size 3 × 3, a Batch Normalization layer (BN), and an interpolation layer with $scale\_factor$ of 8.

### Feature fusion module FF

**Figure 4:**
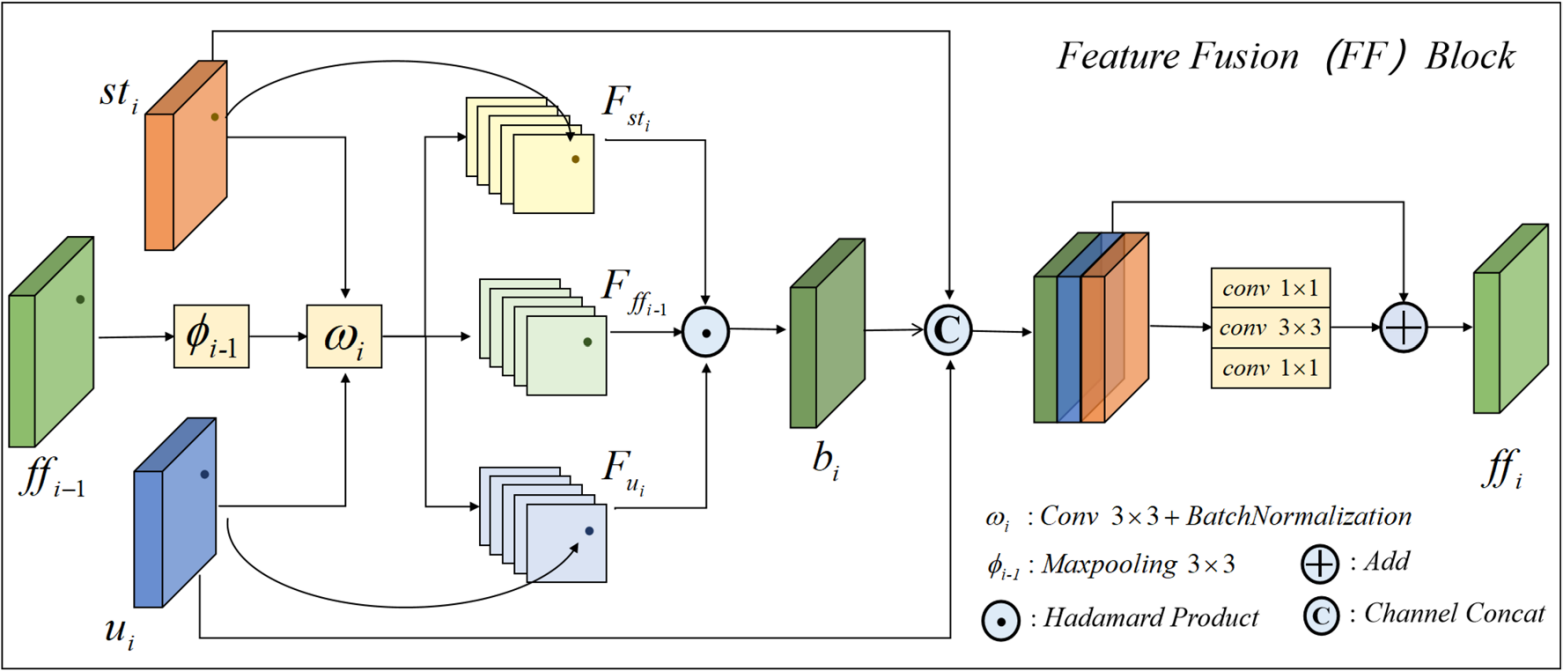
The diagram of the feature fusion module, FF.

We propose an FF module to effectively combine the encoded information extracted from the Swin-Transformer branch and the U-Net branch, as shown in Fig. 4. The module incorporates a multi-modal mechanism and a linear Hadamard product to achieve an interactive fusion of feature information. The multi-modal mechanism fuses the features extracted by the U-Net and Transformer branches under their respective modalities and feeds the intermediate layer information from each modal output to the next layer to emphasize correlation information under different modalities, as shown in the green dashed box in Fig. 1. Specifically, we construct four FF modules for fusing feature maps of different sizes. With the exception of the first FF module, the remaining three FF modules introduce ${ff}_{i-1}$ to achieve feature fusion in different modes. The features map extracted from the Swin-Transformer branch ${st}_{i},i=\mathrm{2,3},4$, the U-Net branch ${u}_{i},i=\mathrm{2,3},4$ and the ${ff}_{i-1}, i=\mathrm{2,3},4$ obtained from the previous FF are refined using convolution operations to obtain ${F}_{{st}_{i}}, { F}_{{ff}_{i-1}}$ and ${F}_{{u}_{i}}$. After that, the features at the same position $l$ are linearly fused (Hadamard product) to obtain the matrix ${b}_{i}$. The first FF module incorporates only ${st}_{1}$ and $u_{1}$. The formulation is shown in Eqs. (9) to (12).

9 $$\begin{array}{*{20}c} {F_{{st_{i} }} \left( {l,st_{i} } \right) = \omega_{ }^{i} \left( {st_{i} } \right) \in R^{M} } \\ \end{array}$$

10 $$\begin{array}{*{20}c} {F_{{u_{i} }} \left( {l,u_{i} } \right) = \omega_{ }^{i} \left( {u_{i} } \right) \in R^{N} } \\ \end{array}$$

11 $$\begin{array}{*{20}c} {F_{{ff_{i - 1} }} \left( {l,ff_{i - 1} } \right) = \omega_{ }^{i} \left( {\phi_{ }^{i - 1} \left( {ff_{i - 1} } \right)} \right) \in R^{P} } \\ \end{array}$$

12 $$\begin{array}{*{20}c} {b_{i} = F_{{st_{i} }} \left( {l,st_{i} } \right) \odot F_{{u_{i} }} \left( {l,u_{i} } \right) \odot F_{{ff_{i - 1} }} \left( {l,ff_{i - 1} } \right) \in R^{M \times N \times P} } \\ \end{array}$$

where $R$ stands for all real numbers, $M, N,P$ is the number of channels in the feature map, ${\omega }^{i},i=\mathrm{1,2},\mathrm{3,4}$ is the convolution operation with a convolution kernel of 3 $\times$ 3, and ${\phi }^{i-1},i=\mathrm{2,3},4$ is the max-pooling layer with filter size 3 $\times$ 3. $\odot$ is the linear Hadamard product operation interacting with feature information at a fine granularity. Finally, the interaction features ${b}_{i}$ and attended features ${st}_{i}$、${u}_{i}$ are concatenated in the channel dimension and passed through a residual block to obtain the fused feature representation ${ff}_{i},i=\mathrm{1,2},\mathrm{3,4}$. The formulation is shown in Eq. (13).

13 $$\begin{array}{*{20}c} {ff_{i} = Residual\left( {Channel\_Concat\left[ {st_{i} ,u_{i} ,b_{i} } \right]} \right)} \\ \end{array}$$

The resulting feature ${ff}_{i}$ effectively captures the global contextual and spatial structure information at the current resolution.

### Decoder construction

**Figure 5:**
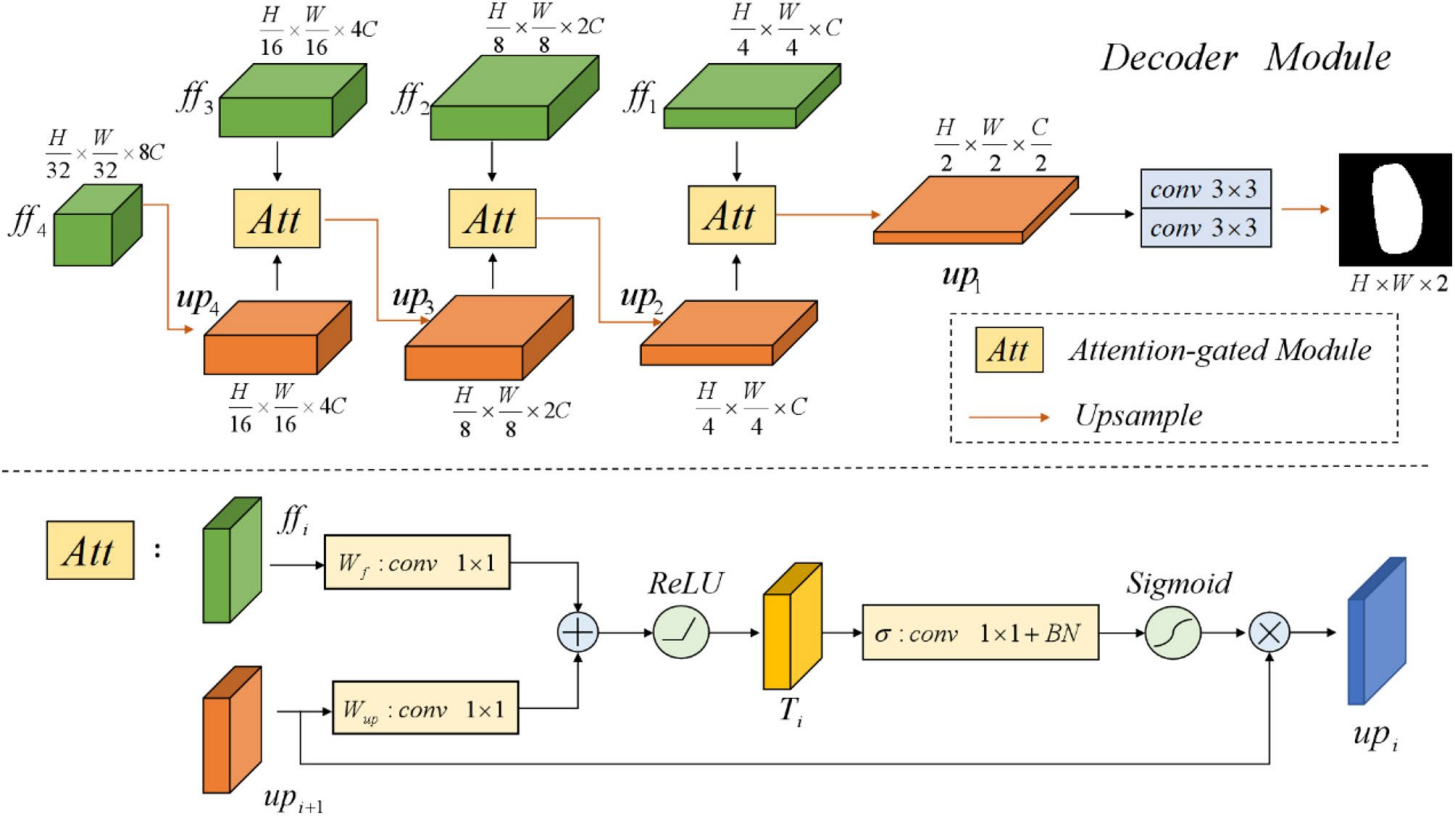
The diagram of the Decoder module.

We pass the multi-scale feature information extracted from the FF module into the Decoder via a skip connection, which is structured to recover the details of the image and output the segmentation results. The overall structure is shown in Fig. 5. In order to suppress irrelevant regions and enable more fine-grained feature interaction fusion, we use the attention-gated module Att^36^ to combine the ${ff}_{i},i=\mathrm{1,2},3$ and the ${up}_{i},i=\mathrm{2,3},4$ recovered by the up-sampling, where ${up}_{4}$ is obtained from ${ff}_{4}$ by linear interpolation with a $scale\_factor$ of 2. In the Att module, we combine the contextual information provided by ${ff}_{i}$ and the spatially detailed information recovered by ${up}_{i+1}$, and map them to the interval {-1,1} by using an activation function to obtain the corresponding weights. Then multiply with ${up}_{i+1}$ to perform adaptive feature modification to incorporate both shallow and deep-level features. The formulation is shown in Eqs. (14) to (15).

14 $$\begin{array}{*{20}c} {T_{i} = ReLU \left[ { W_{f} \left( {ff_{i} } \right) + W_{up} \left( {up_{i + 1} } \right) } \right] i = \left[ {3, 2,1} \right]} \\ \end{array}$$

15 $$\begin{array}{*{20}c} {up_{i} = up_{i + 1} \times Sigmoid\left( { \sigma \left( { T_{i} } \right) } \right)} \\ \end{array}$$

${W}_{f}$ and ${W}_{up}$ are linear transformations of ${ff}_{i}$ and ${up}_{i+1}$ using a convolution with kernel size 1 × 1, and then activated by the ReLU function to obtain the fused feature ${T}_{i}$. $\sigma$ is a normalisation function, consisting of a convolution with kernel size 1 $\times$ 1 and a Batch Normalization (BN) layer. After the combination of the Att module, the feature map ${up}_{1}$ restores its original resolution by a convolution operation and a linear interpolation operation to output the final segmentation map $mask$. The whole formulation is shown in Eqs. (16) to (17).

16 $$\begin{array}{*{20}c} {up_{i} = Att\left[ {up_{i + 1} , ff_{i} } \right], i = 3,2,1} \\ \end{array}$$

17 $$\begin{array}{*{20}c} {mask = Up\left( {conv^{ } \left( {up_{1} } \right)} \right)} \\ \end{array}$$

where $conv$ consists of 3 groups of convolution units, each consisting of a convolution with kernel size 3 $\times$ 3, a Batch Normalization (BN) layer, and the ReLU activation function. $Up$ is linear interpolation with a $scale\_factor$ of 4.

### Loss function

The whole network is trained end-to-end, and the loss value between the segmentation result and the ground truth label is calculated using a cross-entropy loss function. Our labels are divided into two categories, lesion region and background. A pixel-by-pixel comparison of the prediction vector with the ground truth such that $P\left(Y=1\right)=p, P\left(Y=0\right)=1-p$. where $Y=1, 0$ denotes the positive and negative of the label, respectively, and the ground truth is known, i.e., $p = 0, 1$. The probability prediction of the model is calculated by the Softmax function $,$ as follows:

18 $$\begin{array}{*{20}c} {P\left( {\hat{Y} = 1} \right) = \frac{1}{{1 + e^{x} }} = \hat{p}, P\left( {\hat{Y} = 0} \right) = 1 - \frac{1}{{1 + e^{x} }} = 1 - \hat{p}} \\ \end{array}$$

where x is the output of the model and $\hat{Y} = 1, 0$ denotes positive and negative respectively. The losses of the model consist of three parts, the loss $L_{ff}$ obtained via the decoder, the loss $L_{st} , L_{u}$ output by the Swin-transformer and the U-Net via the DFA respectively. The segmentation results obtained through network training are $M_{ff}$, $M_{st}$ and $M_{u}$. The ground truth labels are $G$, all in the range {0,1}. The loss function is shown in Eq. (18).

19 $$\begin{array}{*{20}c} \begin{aligned} L_{i} \left( {G, x} \right) & = - G\log \left( {\frac{1}{{1 + e^{x} }}} \right) + \left( {1 - G} \right)\log \left( {1 - \frac{1}{{1 + e^{x} }}} \right), L_{i} \in \left\{ {L_{ff} ,L_{st} , L_{u} } \right\}, \\ & \quad x \in \left\{ {M_{ff} , M_{st} ,M_{u} } \right\} \\ \end{aligned} \\ \end{array}$$

The final loss value is obtained by multiplying the three-part loss by the corresponding weights and adding them together. The formula for calculating the total loss is shown in Eq. (19).

20 $$\begin{array}{*{20}c} {\begin{array}{*{20}c} {\begin{array}{*{20}c} {L = \alpha \cdot \;L_{ff} + \beta \cdot \;L_{st} + \gamma \cdot \;L_{u} } \\ \end{array} } \\ \end{array} } \\ \end{array}$$

$\alpha ,\beta$ and $\gamma$ are the corresponding weights, which are adjustable hyper-parameters, the specific values set by the experimental results.

## Experiments

### Dataset and evaluation metrics

The gastric cancer images dataset used in this work was from the digestive endoscopy center of General Hospital of the People’s Liberation Army. The study was conducted according to the principles of the Declaration of Helsinki and in accordance with current scientific guidelines. Approval was given by the Ethics Committee of the Chinese People’s Liberation Army General Hospital, and written informed consent was obtained from all subjects and their families.

**Figure 6:**
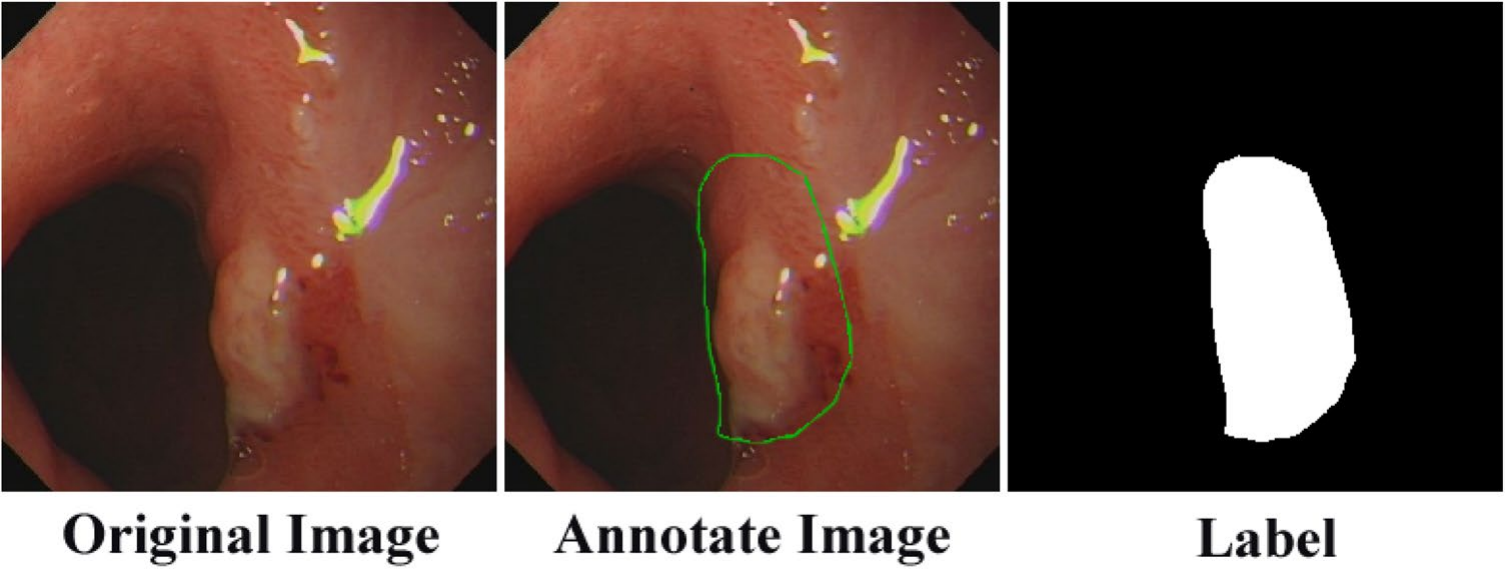
The diagram of the dataset.

The acquired gastric cancer images were manually labeled using $Labelme$ software according to the lesion region marked by the expert. Some of the poor-quality images were removed to ensure the experiment's effectiveness, and 630 pairs of original gastric cancer images and corresponding lesion labeled images were finally selected, as shown in Fig. 6. The images in this dataset were selected from various angles and brightness and at different distances. From 630 pairs of gastric cancer images, 100 pairs of images were randomly selected for testing, and the remaining 530 pairs were used for network training. We resize the images to 224 × 224 to make the dataset images of the same size and meet the network training needs. We augment the training set by flipping the images horizontally and vertically, rotating them at any angle, randomizing hue, saturation, brightness transformations, panning, and zooming to prevent overfitting due to the small amount of data. The final training data were selected as 9360 images. From the enhanced training dataset, 5% (468 images) of the images were randomly selected to form the validation dataset.

In addition, we conducted experiments on the Kvasir-SEG^37^ and CVC-ClinicDB^38^ datasets to evaluate the effectiveness and generalization performance of the proposed method in this paper. The Kvasir-SEG dataset is the first for gastrointestinal disease identification and contains 1000 images of polyp lesions and their corresponding masks. The CVC-ClinicDB dataset includes 612 high-resolution images from 31 colonoscopies. The original images were in "tif" format, which we converted to "png" format. We cropped the images uniformly to 224 × 224 large to fit the network training requirements and divided the training set, validation set, and test set according to the ratio of 8:1:1.

We used the $Python$ and the $PyTorch$ framework to build the experimental environment, and the $GTX3080 GPU$ device to complete the network training. The experiment set the $epoch$ size to 300, the $batch size$ to 16, and the $Adam$ optimizer to update the network weights, setting the $Learning rate$ to ${1e}^{-3}$ and the $weight decay$ to ${1e}^{-4}$. We used a pre-trained on $Image-1K$ mode $swin\_tiny\_patchh4\_window7\_224$ to speed up the network training. We evaluate the segmentation performance of the proposed method, namely IOU, Dice, Accuracy (ACC), Recall (RE), Precision (PR), Specificity (SP) and F1-Score. Evaluation metrics are defined as Eqs. (21) to (27). Where TP, TN, FP, and FN show the true positive, true negative, false positive, and false negative samples, respectively.

21 $$\begin{array}{*{20}c} {\begin{array}{*{20}c} {IOU = \frac{TP}{{TP + FP + FN}} } \\ \end{array} } \\ \end{array}$$

22 $$\begin{array}{*{20}c} {Dice = \frac{2TP}{{2TP + FP + FN}}} \\ \end{array}$$

23 $$\begin{array}{*{20}c} {Accuracy = \frac{TP + TN}{{TP + FP + TN + FN}}} \\ \end{array}$$

24 $$\begin{array}{*{20}c} {Recall = \frac{TP}{{TP + FN}}} \\ \end{array}$$

25 $$\begin{array}{*{20}c} {Precision = \frac{TP}{{TP + FP}}} \\ \end{array}$$

26 $$\begin{array}{*{20}c} {Specificity = \frac{TN}{{TN + FP}}} \\ \end{array}$$

27 $$\begin{array}{*{20}c} {F1 - Score = 2 \times \frac{Precision \times Recall}{{Precision + Recall}}} \\ \end{array}$$

### Ablation experiments results

We use ablation experiments to investigate the effectiveness of the DFA module and the fusion Transformer and U-Net approaches. Experiments “U-Net” and “ST” used the original U-Net and Swin-Transformer to segment the gastric cancer lesion region. Experiments “U-Net + DFA” and “ST + DFA” replaced the decoder part of the U-Net and Swin-Transformer with the DFA module proposed in this study to evaluate its effectiveness. Experiment “Fusion + FF” uses the original U-Net and Swin-Transformer structures and fuses the feature information output from both using the FF module to verify the effectiveness of the fusion approach. Experiment “Ours” is an experiment on the model proposed in this paper. Table 1 shows the average and standard deviation of the evaluation metrics for the 100 test images, and Table 2 utilizes the “Params” to characterize the number of parameters for each model.

**Table 1:** Comparison of ablation experiment results.

Method	IOU	Dice	ACC	RE	PR	SP	F1-Score
U-Net	0.641 $\pm$ 0.140	0.778 $\pm$ 0.097	0.876 $\pm$ 0.045	0.762 $\pm$ 0.163	0.848 $\pm$ 0.209	0.851 $\pm$ 0.078	0.803 $\pm$ 0.114
ST	0.685 $\pm$ 0.117	0.810 $\pm$ 0.054	0.893 $\pm$ 0.011	0.799 $\pm$ 0.136	0.827 $\pm$ 0.073	0.931 $\pm$ 0.029	0.810 $\pm$ 0.042
U-Net + DFA	0.729 $\pm$ 0.109	0.841 $\pm$ 0.083	0.909 $\pm$ 0.031	0.833 $\pm$ 0.142	0.854 $\pm$ 0.081	0.939 $\pm$ 0.053	0.843 $\pm$ 0.087
ST + DFA	0.739 $\pm$ 0.111	0.847 $\pm$ 0.046	0.912 $\pm$ 0.012	0.832 $\pm$ 0.101	0.866 $\pm$ 0.056	0.943 $\pm$ 0.023	0.849 $\pm$ 0.049
Fusion + FF	0.745 $\pm$ 0.083	0.854 $\pm$ 0.035	0.916 $\pm$ 0.017	0.856 $\pm$ 0.071	0.847 $\pm$ 0.051	0.936 $\pm$ 0.019	0.852 $\pm$ 0.028
Ours	**0.813** $\pm$ **0.075**	**0.895** $\pm$ **0.032**	**0.940** $\pm$ **0.011**	**0.888** $\pm$ **0.066**	**0.907** $\pm$ **0.042**	**0.962** $\pm$ **0.008**	**0.897** $\pm$ **0.022**

*“ST” indicates the Swin-Transformer model, and “Fusion” indicates the fusion of two branches, “$\mathrm{DFA}$” is the deep feature aggregation decoder, and “FF” is the feature fusion module. Bold characters indicate the best performance.

**Table 2:** Comparison of model parametric quantities.

	U-Net	ST	U-Net + DFA	ST + DFA	Fusion + FF	Ours
Params	17.27 M	28 M	12.20 M	21.97 M	57.09 M	53.90 M

**Figure 7:**
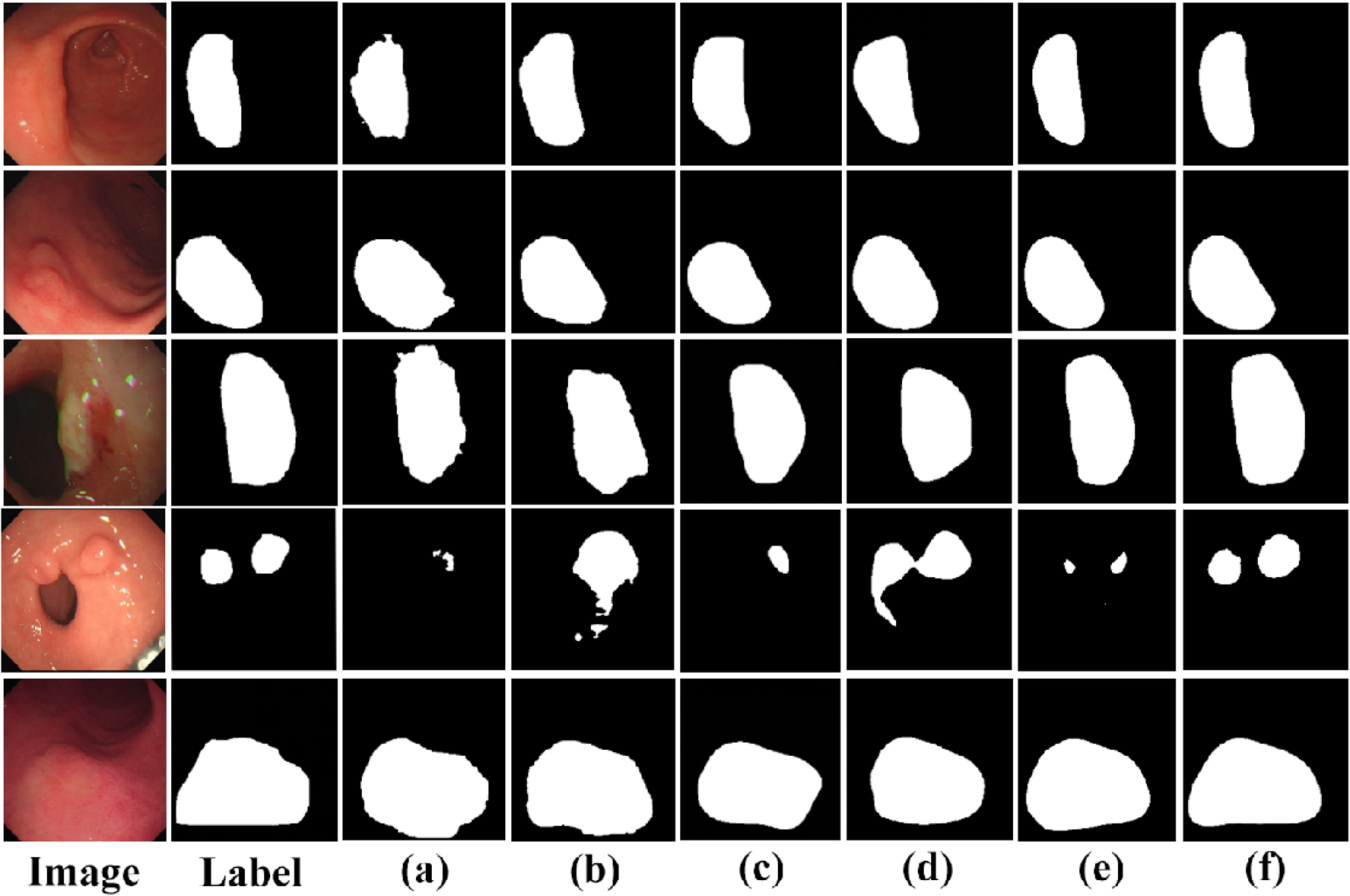
Segmentation result of gastric cancer images. Image is the original gastric image; Label is the ground truth label; (**a**) to (**e**) correspond to the lesion segmentation results obtained from the “U-Net”, “ST”, “UNet + DFA”, “ST + DFA” and “Fusion + FF” in Table 1, respectively. Where (**f**) is the segmentation result obtained from the model proposed in this paper.

**Figure 8:**
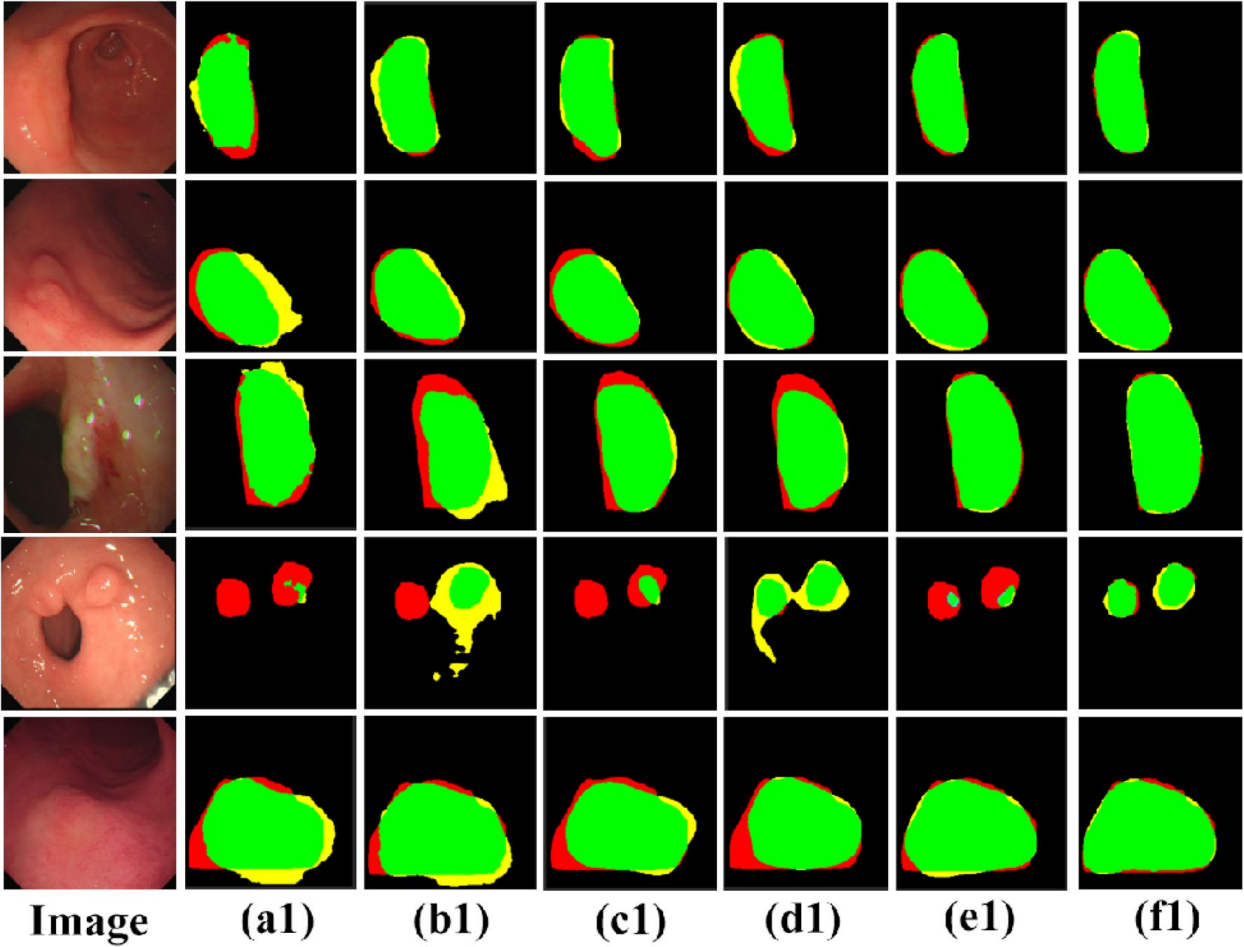
Comparison of true labels and segmentation results. Red represents the ground truth label, yellow represents the predicted result, and the intersection of both is green.

As seen in Table 1, the results are the most unsatisfactory when using only the U-Net or the Swin-Transformer for image segmentation, with IOU coefficients reaching only 64.1% and 68.5%. We replaced the decoders in U-Net and Swin-Transformer with DFA modules, i.e., “UNet + DFA” and “ST + DFA”, in Table 1. The segmentation results showed a significant improvement, with the U-Net IOU coefficient reaching 72.9%, an improvement of 8.8%, and the Swin-Transformer IOU coefficient reaching 73.9%, an improvement of 5.4%. As seen in Table 2, the use of the DFA module effectively reduces the number of parameters and decreases the complexity of the model compared to the original decoder. After that, we used the FF module to fuse the two branches, the IOU coefficient reached 74.5%, a 6% improvement over the best result of both, proving that fusing two branches using the FF module yields better segmentation results. Using the FF module and DFA module, the IOU coefficient of the fused network reached 81.3%, an improvement of 6.8% compared to the best results above. The best performance in all other evaluation metrics demonstrates the effectiveness of the method proposed in this paper. It is further demonstrated that fusing Swin-Transformer and U-Net can produce better segmentation results. The segmentation results obtained for several network models are shown in Figs. 7 and 8.

As can be seen from Figs. 7 and 8, the segmentation result of (f) is closer to the ground truth labels, once again proving the effectiveness of our proposed method. (e) shows the segmentation results generated by fusing Swin-Transformer and U-Net using the FF module. It can be seen that lesion localization is more accurate than using only Swin-Transformer, and it also focuses on global information and gives better results in the presence of multiple lesions than using only the U-Net. (c) and (d) are the segmentation results obtained by using the DFA. It can be seen that the edges are more evident than in (a) and (b) because the RFB module increases the receptive field while effectively suppressing interference information. Besides, (b) and (d) are segmentation results generated using Swin-Transformer as the backbone. It can be found that the Swin-Transformer architecture pays attention to discontinuous lesion regions compared to the generated results obtained from (a) and (c) using U-Net as the backbone. The result proves that the Transformer is better focused on extracting global contextual information and performs better in modeling explicit long-range relations. The direct comparison between the ground truth labels and the segmentation results in Fig. 8 provides a more intuitive indication of the quality of the segmentation results. It shows that the segmentation results obtained by our proposed model are closer to the actual labels.

### Comparative experiments results

**Figure 9:**
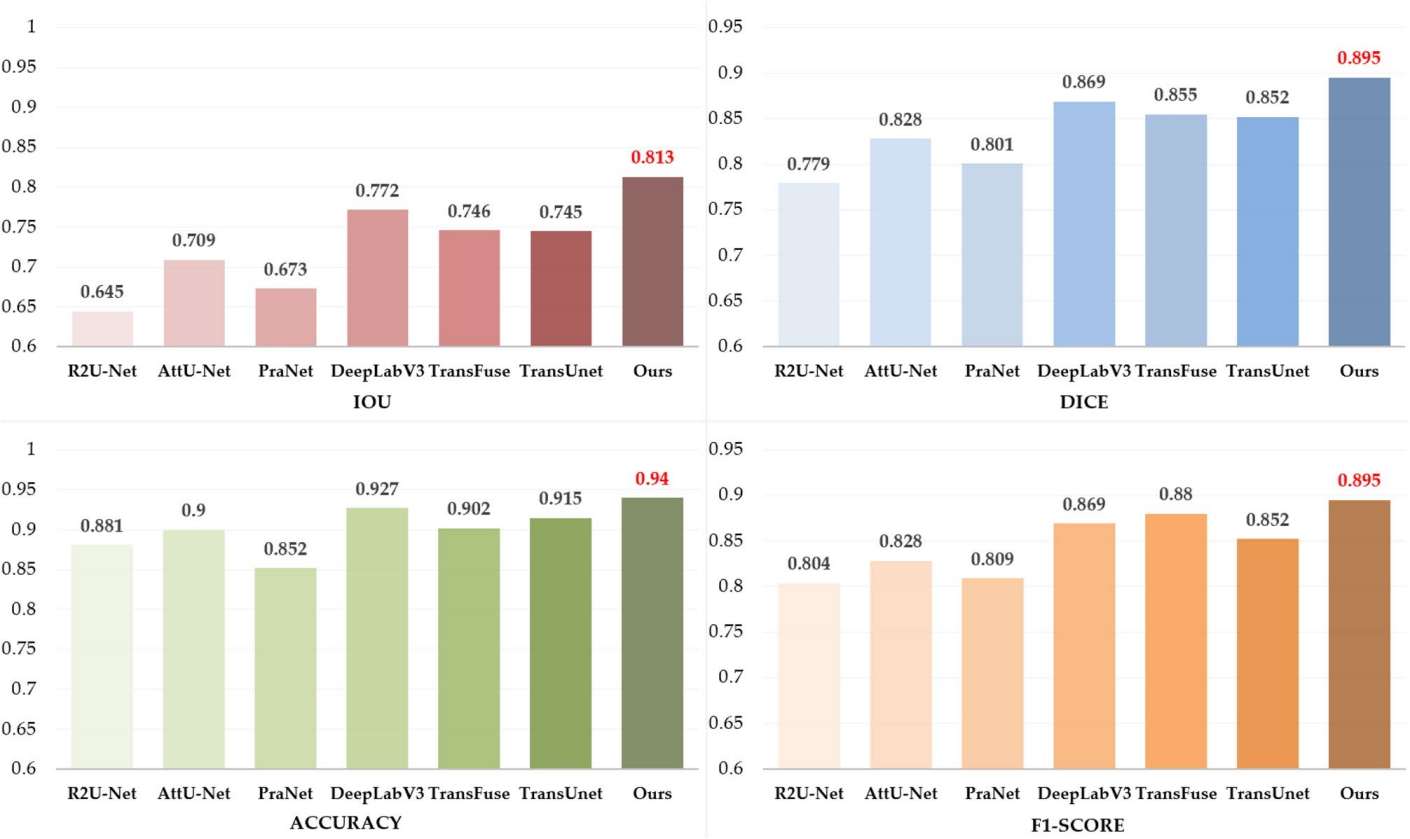
Comparison results of evaluation indicators.

**Figure 10:**
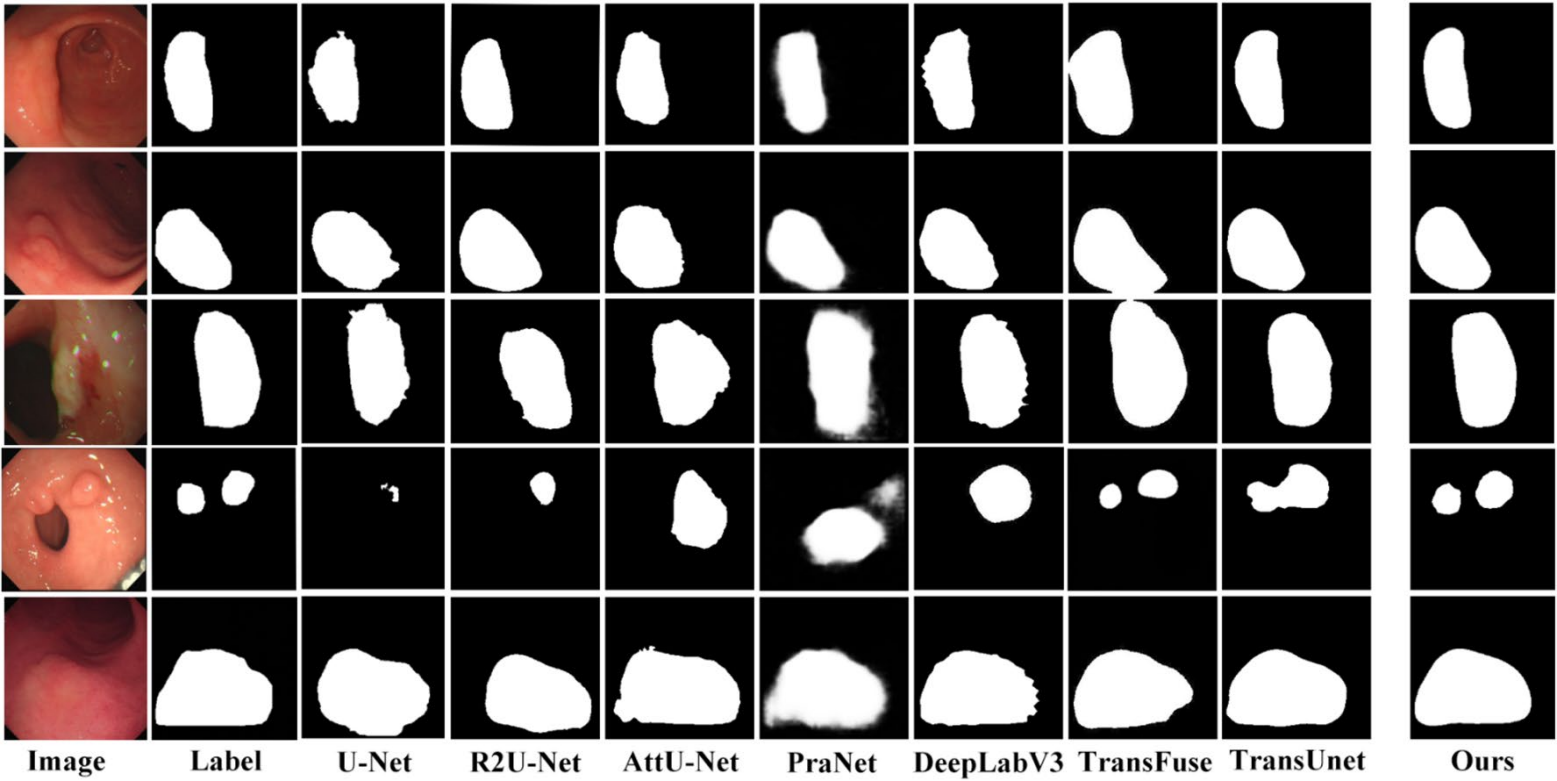
Segmentation results compared with other models.

In this paper, we also compare our proposed model with several previous image segmentation methods, and the average results are shown in Table 3. For a fair comparison, all experiments use the same data pre-processing, pretraining parameters, and evaluation metrics. Compared with R2U-Net, AttU-Net, PraNet, and DeepLabV3, our IOU indexes improved by 16.8%, 10.4%, 14%, and 4.1%, and the other performance indexes were all optimal values. Compared with TransUNet and TransFuse, which also use the combination of CNNs and Transformers, the IOU indexes improved by 6.7% and 6.8%, which proves that our proposed method is more effective for gastric cancer lesion segmentation. The histogram in Fig. 9 provides a more precise visual comparison of the results of our model with those of other leading models. Figure 10 shows the segmentation results obtained by each model on our dataset. The combination of Figs. 9 and 10 again demonstrates that our model performs well in lesion segmentation of gastric cancer images, yielding high-quality segmentation results with the best segmentation performance.

**Table 3:** Comparative experimental results with other methods.

Method	IOU	Dice	ACC	RE	PR	SP	F1-Score
U-Net^8^	0.641 $\pm$ 0.140	0.778 $\pm$ 0.097	0.876 $\pm$ 0.045	0.762 $\pm$ 0.163	0.848 $\pm$ 0.209	0.851 $\pm$ 0.078	0.803 $\pm$ 0.114
R2U-Net^39^	0.645 $\pm$ 0.185	0.779 $\pm$ 0.101	0.881 $\pm$ 0.046	0.759 $\pm$ 0.189	0.852 $\pm$ 0.141	0.883 $\pm$ 0.066	0.804 $\pm$ 0.109
AttU-Net^40^	0.709 $\pm$ 0.134	0.828 $\pm$ 0.071	0.900 $\pm$ 0.021	0.832 $\pm$ 0.073	0.831 $\pm$ 0.123	0.931 $\pm$ 0.030	0.831 $\pm$ 0.049
PraNet^41^	0.673 $\pm$ 0.171	0.801 $\pm$ 0.107	0.852 $\pm$ 0.057	0.789 $\pm$ 0.176	0.831 $\pm$ 0.135	0.853 $\pm$ 0.075	0.809 $\pm$ 0.108
DeepLabV3^42^	0.772 $\pm$ 0.083	0.869 $\pm$ 0.035	0.927 $\pm$ 0.017	0.870 $\pm$ 0.085	0.871 $\pm$ 0.051	0.946 $\pm$ 0.019	0.870 $\pm$ 0.028
TransFuse^17^	0.746 $\pm$ 0.077	0.855 $\pm$ 0.033	0.902 $\pm$ 0.010	0.871 $\pm$ 0.087	0.891 $\pm$ 0.070	0.882 $\pm$ 0.021	0.880 $\pm$ 0.065
TransUnet^18^	0.745 $\pm$ 0.082	0.852 $\pm$ 0.031	0.915 $\pm$ 0.016	0.849 $\pm$ 0.091	0.858 $\pm$ 0.056	0.940 $\pm$ 0.018	0.852 $\pm$ 0.035
Ours	**0.813** $\pm$ **0.075**	**0.895** $\pm$ **0.032**	**0.940** $\pm$ **0.011**	**0.888** $\pm$ **0.066**	**0.907** $\pm$ **0.042**	**0.962** $\pm$ **0.008**	**0.897** $\pm$ **0.022**

*Bold characters indicate the best performance.

### Validation experiments on public datasets

In our work, we also conducted experiments on the Kvasir-SEG and CVC-ClinicDB datasets to evaluate the generalization performance of the models. All experiments use the same experimental environment, data pre-processing methods, and pre-training parameters. We used IOU, Dice, ACC, RE, and PR to evaluate the experimental results, and the average results are shown in Table 4.

**Table 4:** Experimental results in comparison with other algorithms.

Method	IOU	Dice	ACC	RE	PR
Kvasir-SEG					
U-Net	0.749	0.821	0.923	0.817	0.825
U-Net + +	0.752	0.825	0.927	0.824	0.831
DeepLabV3	0.801	0.876	0.956	**0.923**	0.841
PraNet	**0.835**	**0.896**	0.973	0.915	0.885
TransFuse	0.784	0.873	0.957	0.898	0.862
TransUnet	0.796	0.884	0.962	0.905	0.873
Ours	0.823	0.892	**0.975**	0.917	**0.909**
CVC-ClinicDB					
U-Net	0.727	0.825	0.923	0.827	0.831
U-Net + +	0.734	0.837	0.935	0.841	0.827
DeepLabV3	0.748	0.849	0.968	0.879	0.836
PraNet	0.849	0.899	0.982	**0.936**	**0.896**
TransFuse	0.765	0.852	0.978	0.885	0.843
TransUnet	0.837	**0.909**	0.979	0.895	0.874
Ours	**0.851**	0.893	**0.985**	0.907	0.889

*Bold characters indicate the best performance.

**Figure 11:**
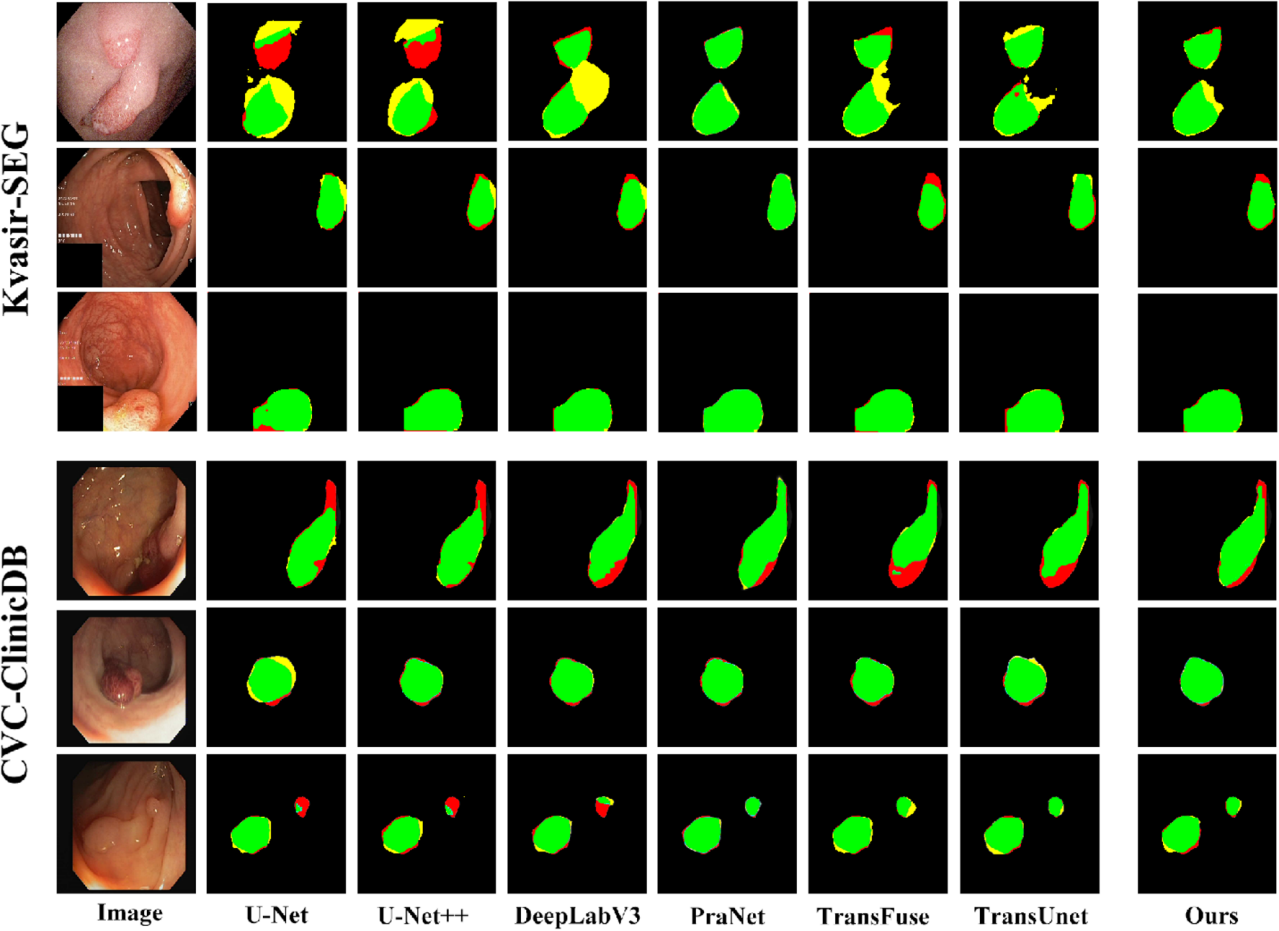
Comparison of the ground truth labels and segmentation results of the colon polyp datasets.

As can be seen from Table 4, on the Kvasir-SEG dataset, the best performing IOU and Dice coefficients are PraNet, but our model differs from it by only 1.2% and 0.04%; the best recall is DeepLabV3, and we differ from it by only 0.6%, and our proposed model is the optimal performance in terms of ACC and PR indexes. On the CVC-ClinicDB dataset, our proposed model achieves IOU and ACC of 85.1% and 98.5%, optimal values; Dice of 89.3%, which differs from the best-performing TransUnet by 1.6%; Re and PR differ from the best-performing model by 2.9% and 1.3%. The experimental results show that our model performs well on the publicly available colon polyp dataset, further demonstrating the excellent generalization performance of the model for the segmentation of other endoscopic lesion regions. Figure 11 shows the segmentation results obtained for each model on the Kvasir-SEG and CVC-ClinicDB datasets that overlap with the ground truth labels. Red represents the ground truth label, yellow represents the predicted result, and the intersection of both is green. The results show that our proposed model is close to the actual segmentation results and produces high-quality results.

## Discussion

The total loss function $L=\alpha \cdot {L}_{ff}+\beta \cdot {L}_{st}+\gamma {\cdot L}_{u}$, and the weights $\alpha ,\beta$ and $\gamma$ of its three parts need to be determined by the experimental results. $\alpha ,\beta$ and $\gamma$ range between [0, 1], and $\alpha +\beta +\gamma =1.$ In Table 1, we have experimentally confirmed that the segmentation results obtained by fusing Swin-transformer and U-Net are satisfactory, and that the segmentation results obtained by using only Swin-Transformer are better than those obtained by using only U-Net. Therefore, on the initial value setting, we define $\alpha =0.5,\beta =0.3,\gamma =0.2.$

Table 5 shows that $\alpha =0.5,\beta =0.2,\gamma =0.3$ give the best results. It can be found from the experiments that increasing the U-Net loss weights gives better results than increasing the Swin-Transformer loss weights, which is contrary to our proposed hypothesis. However, Table 1 shows that “U-Net + DFA” is 8.8% better than the U-Net segmentation, “ST + DFA” is 5.4% better than the Swin-Transformer segmentation, and “ST + DFA” is only 0.1% better than “U-Net + DFA”. The result demonstrates that the DFA module impacts on the segmentation results and works more effectively than the U-Net in dealing with the gastric cancer image segmentation problem. Therefore, in our experiments, we set $\alpha =0.5,\beta =0.2,\gamma =0.3.$

**Table 5:** Comparison experiments of loss function weight values.

$\alpha ,\beta ,\gamma$	Loss	IOU	Dice	ACC	RE	PR	SP
0.5,0.3,0.2	0.178	0.771	0.870	0.927	0.862	0.884	0.873
0.6,0.2,0.2	0.199	0.747	0.854	0.919	0.845	0.866	0.855
0.5,0.2,0.3	**0.139**	**0.813**	**0.895**	0.940	**0.888**	0.907	**0.897**
0.5,0.1,0.4	0.145	0.804	0.887	**0.942**	0.882	0.903	0.892
0.4,0.2,0.4	0.167	0.781	0.873	0.924	0.875	0.891	0.883
0.6,0.1,0.3	0.141	0.808	0.893	0.941	0.865	**0.925**	0.894

*Bold characters indicate the best performance.

**Figure 12:**
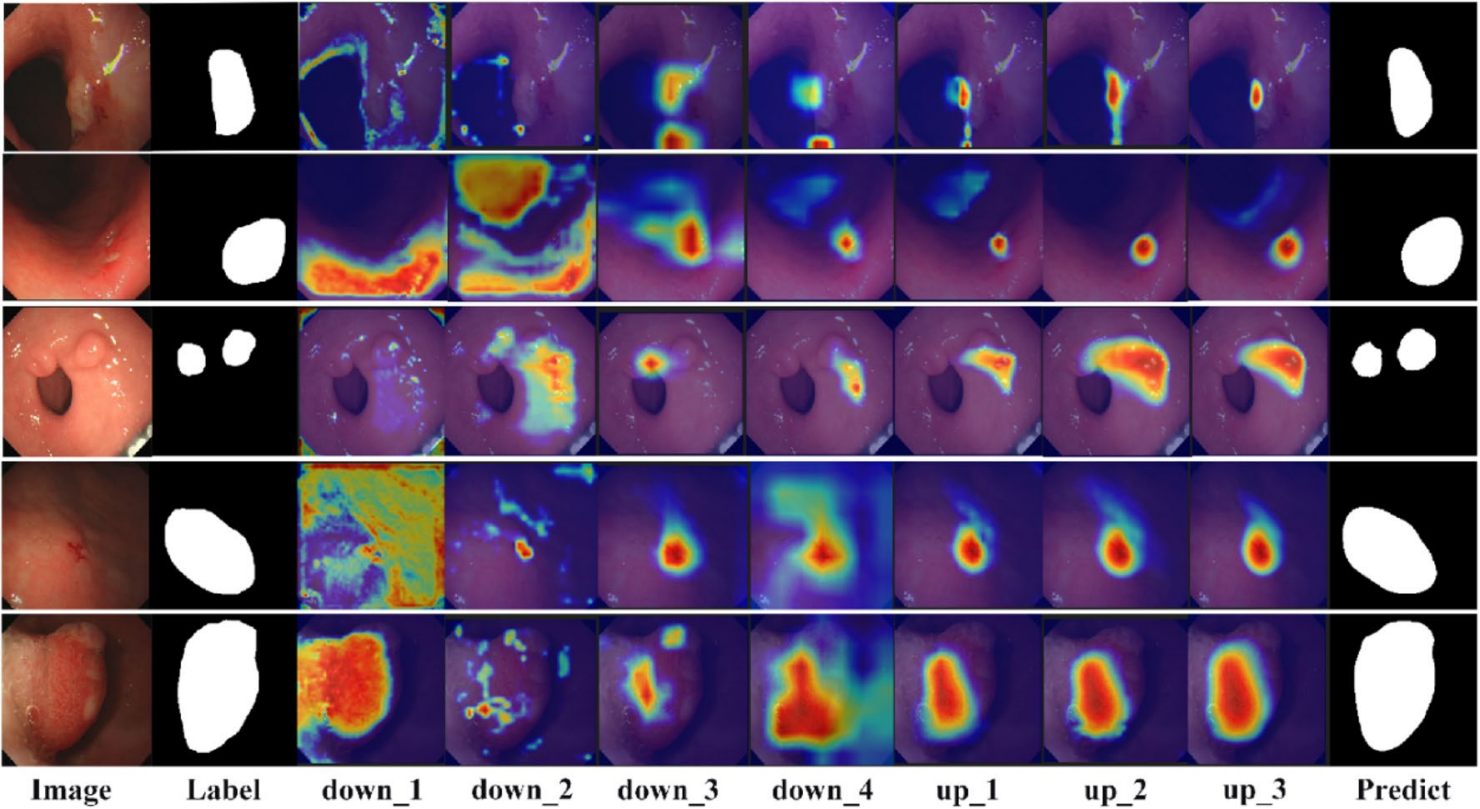
The diagram of the Grad-CAM visualization process.

For a more concrete visualization of the entire area of interest of the model, a heat map was created using Grad-CAM visualization. Grad-CAM^43^ uses the network back propagation gradient to calculate the weights of each channel of the feature map to obtain the heat map. Our model focuses on the regions of interest for feature layers $down\_1$ to $down\_4$, which use the FF module for feature fusion during down-sampling, and feature layers $up\_1$ to $up\_3$, which recover resolution during up-sampling. The blue and red colors on Grad-CAM indicate lower and higher activation values, respectively. The specific visualization results are shown in Fig. 12. The down-sampling process gradually focuses the network from low-level to high-level semantic features and can pinpoint the location of the lesion. During up-sampling to recover resolution, the model further incorporates low-level semantic features passed through the skip connection to make accurate predictions about the location of the lesion. Using Grad-CAM to visualize the whole process once again proves that our segmentation model can produce more accurate segmentation results.

## Conclusions

In this paper, we proposed a Dual-Branch Hybrid Network that effectively fuses the Swin-Transformer and the U-Net for lesion segmentation of gastric cancer images. We built the Deep Feature Aggregation Decoder DFA to replace the original decoder structure of the network, effectively reducing the complexity of the model and pinpointing the lesion regions. Besides, we used the FF module to fuse the advantageous features extracted by the U-Net and Transformer, compensating for the lack of global contextual information obtained by the former and the inadequate capture of spatially detailed information in the latter. Our experiments also demonstrated that the FF and DFA modules positively affect the segmentation results. We computed a three-part loss to iteratively train the network, making the segmentation results closer to the ground truth labels. In addition, the region of interest for the entire network model was visualized using Grad-CAM, reflecting side by side that our segmentation network is realistic and practical. Performance indicators showed that our model achieves a very satisfactory 81.3% IOU, 89.5% Dice, and 94.0% accuracy in the segmentation of the lesion region, achieving optimal results in several evaluation metrics and outperforming existing segmentation models. The result of the model was closer to the manual segmentation standard for lesions in gastric cancer images. Our experimental results show that the IOU can still be further improved. In the image segmentation task, the fuzzy labeling of the lesion boundary region with the background region leads to a poor learning ability of the model at the boundary location, which explains the relatively low IOU. In future work, we will improve the IOU by enhancing the ability to extract features from boundary regions. Meanwhile, we need to improve generalization performance to promote it in other medical segmentation domains.

## Data Availability

The datasets used and/or analyzed during the current study available from the corresponding author on reasonable request.
